# Estimation of the synaptic conductance in a McKean-model neuron

**DOI:** 10.1186/1471-2202-16-S1-P251

**Published:** 2015-12-18

**Authors:** Antoni Guillamon, Rafel Prohens, Antonio E Teruel, Catalina Vich

**Affiliations:** 1 Dept. of Applied Mathematics I, EPSEB, Universitat Politècnica de Catalunya, 08028 Barcelona, Spain; 2 Dept. of Mathematics and Computer Science, Universitat de les Illes Balears, 07122, Palma, Spain

## 

**Figure 1:**
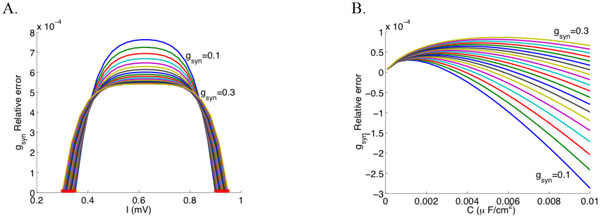
**Goodness of fit of the synaptic conductance parameter**. Panel A represents the relative error versus the applied current for a fixed value of C = 10^-4^. Red points represent the values of I_1 _(left points) and I_2 _(right points) for each g_syn_. Panel B represents the relative error versus the membrane capacitance for a fixed value of I=I_1_+10^-3^. In both panels, the different color traces correspond to different values of g_syn _equally spaced from 0.1 to 0.3. The rest of parameters are fixed as ***a***=0.25, v_0_=0, w_0_=0, γ=0.5, v_syn_=0.25+***a ***/2.

Estimating the synaptic conductances impinging on a single neuron directly from its membrane potential is one of the open problems to be solved in order to understand the flow of information in the brain. Despite the existence of some computational strategies that give circumstantial solutions ([1-3] for instance), they all present the inconvenience that the estimation can only be done in subthreshold activity regimes. The main constraint to provide strategies for the oscillatory regimes is related to the nonlinearity of the input-output curve and the difficulty to compute it. In experimental studies it is hard to obtain these strategies and, moreover, there are no theoretical indications of how to deal with this inverse non-linear problem. In this work, we aim at giving a first proof of concept to address the estimation of synaptic conductances when the neuron is spiking. For this purpose, we use a simplified model of neuronal activity, namely a piecewise linear version of the Fitzhugh-Nagumo model, the McKean model ([4], among others), which allows an exact knowledge of the nonlinear f-I curve by means of standard techniques of non-smooth dynamical systems. As a first step, we are able to infer a steady synaptic conductance from the cell's oscillatory activity. As shown in Figure 1, the model shows the relative errors of the conductances of order C, where C is the membrane capacitance (C<<1), notably improving the errors obtained using filtering techniques on the membrane potential plus linear estimations, see numerical tests performed in [5].
